# Rapid ^13^C Solid-State Quantitative NMR Method for Multiple Physical and Chemical Analyses of Cocoa-Based Products: Proof of Concept

**DOI:** 10.1021/acs.analchem.5c04122

**Published:** 2025-09-07

**Authors:** Thais Juliana Tobias, Priscilla Efraim, Tiago Bueno de Moraes, Luiz Alberto Colnago

**Affiliations:** † São Carlos Institute of Chemistry, 28133 University of São Paulo (USP), Av. Trabalhador São-carlense 400, São Carlos, São Paulo 13660-970, Brazil; ‡ Department of Food Engineering and Technology, 28132 University of Campinas (UNICAMP), St. Monteiro Lobato 80, Campinas, São Paulo 13083-862, Brazil; § Department of Biosystems Engineering, Luiz de Queiroz College of Agriculture, University of São Paulo (USP), Av. Pádua Dias 11, Piracicaba, São Paulo 13418-900, Brazil; ∥ 564899 Embrapa Instrumentation, Rua XV de Novembro 1452, São Carlos, São Paulo 13560-970, Brazil

## Abstract

Chocolates and other cocoa products represent a multibillion-dollar industry that has faced significant price increases, largely due to a surge in cocoa plant diseases linked to climate change. One potential solution for mitigating cocoa prices involves the use of cocoa butter equivalents, substitutes, or replacers. Consequently, a rapid method for simultaneously determining multiple properties of cocoa derivatives can serve as a valuable tool for research and development of new products, quality control, and regulatory agencies to ensure compliance with cocoa product standards. In this context, a rapid quantitative ^13^C solid-state NMR (^13^C qSS-NMR) approach has been developed to assess various physical and chemical properties of cocoa products in a single measurement. In this study, ^13^C qSS-NMR spectra were obtained by directly exciting the ^13^C transitions using 90° or low-flip-angle pulses, along with high-power decoupling and 3 kHz magic-angle sample spinning (MAS). The areas and chemical shifts of the signals at approximately 34.5 and 30 ppm, assigned to the solid and liquid phases of triacylglycerides (TAGs), were utilized to determine the solid fat content (SFC) and the polymorphic forms of TAGs. Additionally, the sucrose content in chocolates was estimated by the ratio of the sucrose signals between 103 and 82 ppm and the TAGs signals. The SFC values were consistent with those obtained by standard methods. The ^13^C SS-NMR approach also holds promise for measuring other cocoa product properties, such as isothermal crystallization, and it can be applied to assess similar properties in other fat-based food products.

## Graphical abstract

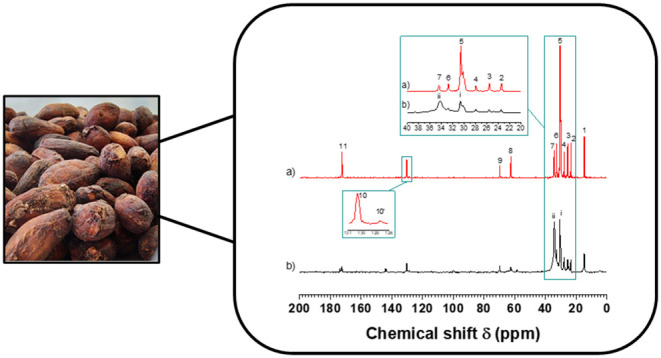

## Introduction

^1^H quantitative nuclear magnetic resonance (qNMR) spectroscopy of liquids or solutions, commonly referred to as solution-state NMR, has been widely employed to quantify the concentration of single or multiple organic analytes across various metabolomics, natural products, food science, forensics, liquid fuels, environmental, and pharmaceutical studies.^1–7^

Conversely, NMR analysis of organic compounds in the solid state has been rarely performed using ^1^H nuclei due to the strong homonuclear dipolar interactions, which require specialized probes and sophisticated pulse sequences.^6,8^ Therefore, the analysis of organic compounds in the solid state has predominantly utilized ^13^C NMR (^13^C SS-qNMR) spectroscopy, given that carbon atoms are prevalent in organic molecules.^7,9^ The advantages of ^13^C SS-NMR compared to ^1^H SS-NMR include greater chemical shift dispersion, leading to higher resolution as well as the ability to easily suppress heteronuclear dipolar interactions and chemical shift anisotropy effects through high-power decoupling (DEC) and moderate magic angle spinning (MAS) frequencies, respectively.^6,7,9^ However, ^13^C SS-NMR has notable disadvantages relative to ^1^H SS-NMR, such as longer measurement time due to its lower isotopic natural abundance, lower magnetogyric ratio, and very long longitudinal relaxation time, which can range from a fraction of a second to several minutes. ^9,10^ For instance, quantitative ^13^C NMR measurements of solid lignin derivatives required nearly 6 days, employing 90° pulses, a 5*T*_1_ (250 s) recycle delay, and 2000 scans.^10^

To reduce measurement time, solid-state ^13^C spectra are typically acquired using a cross-polarization (CP) pulse sequence. This method enhances the signal-to-noise ratio (SNR) by up to four times and utilizes a considerably shorter *T*_1_ relaxation time for ^1^H nuclei.^5,6,9,10^ However, the efficiency of cross-polarization (CP) is highly dependent on the strength of the dipolar interaction, which is influenced by the ^1^H–^13^C internuclear distance and the molecular dynamics.^5,6^

For heterogeneous materials that contain both solid and liquid phases, such as cocoa products, the cross-polarization (CP) sequence effectively enhances signals primarily from the solid phase.^11^ Consequently, the signal of cocoa products in the liquid state is either very weak or absent in CP measurements.^11^ As a result, the CP sequence is not suitable for the quantitative analysis of both the liquid and solid phases in these heterogeneous products. To achieve accurate quantitative ^13^C qSS-NMR measurements, direct excitation of the ^13^C transition is necessary combined with high-power decoupling and MAS. This procedure is known as HPDEC or direct polarization magic angle spinning (DPMAS).^9^

Similar to quantitative NMR (qNMR) in solution, when using a 90° flip angle (β), the repetition time (essentially the recycle delay in qSS-NMR experiments) should be approximately five times the longitudinal relaxation time *T*_1_ (5*T*_1_) of the signals involved in the measurements.^10^ Conversely, the recycle delay can be significantly shorter than *T*_1_ when the excitation is performed using the Ernst angle or other low flip angles.^12^ While the use of a low flip angle decreases the signal intensity in each scan, it is possible to enhance the SNR by conducting a significantly larger number of scans per unit of time.^12^

In this study, we demonstrate the effective application of ^13^C qSS-NMR, employing the HPDEC sequence with a value of β = 90° or lower, to determine various physical and chemical properties of cocoa products that encompass both solid and liquid phases, all within a single experiment lasting approximately 1 h.

Chocolates and other cocoa-related products represent a multibillion-dollar industry^13^ that is predominantly dependent on cocoa beans, which have experienced a significant price surge in the global market in recent years. This increase is primarily driven by rising demand coupled with a decline in supply due to diseases affecting cocoa crops.^14–18^ These challenges may lead to further price hikes for cocoa beans, which could, in turn, result in increased instances of fraud and adulteration in both raw and processed cocoa products.^19^

An alternative approach to lowering cocoa product prices involves the use of cocoa butter substitutes (CBSs), cocoa butter replacers (CBRs), or cocoa butter equivalents (CBEs).^20–24^ In European countries, the addition of up to 5% of CBEs is permissible.^25^ Consequently, the ^13^C qSS-NMR analysis emerges as a viable analytical method for research and development of new cocoa-based products, as well as for industrial quality control (QC) and quality assurance (QA) by regulatory agencies to ensure compliance with standards for cocoa products.

## Materials and Methods

The cocoa products, which include cocoa butter (CB), cocoa liquor (CL), and chocolates, were derived from cocoa trees cultivated in the Brazilian states of Pará (PA), Bahia (BA), and Espírito Santo (ES), as well as from selected commercial sources.^26^ A total of six samples of CB were obtained from cocoa beans: three sourced in the Espírito Santo State (CB_1 to CB_3), two from a commercial supplier (CB_4 and CB_5), and one from Bahia State (CB_6). The cocoa liquors (CL) were obtained from cocoa beans from the Pará State (CL_1), from a commercial supplier (CL_2), and from the Bahia State (CL_3). Dark chocolates (DC) were formulated with cocoa contents of 70% and 60%, using cocoa beans from the states of PA (DC60_2) and BA (for all other DC60 and DC70 samples), while DC40 was sourced as a commercial product. Milk chocolates (MC_1–MC_5) were produced using commercial CB_5, CL_2, and powdered milk with a 4% fat content, whereas MC_6 was prepared using cocoa liquor (CL_3) and powdered milk with a 5.2% fat content.

### Determination of SFC by ^1^H TD-NMR Using ISO Protocols

The values of SFC for the various samples were determined using both ISO protocols: the direct method (ISO 8292-1)^27^ and the indirect method (ISO 8292-2).^28^ Measurements were performed using a Minispec mq-20 spectrometer (Bruker, Germany), which is equipped with a 0.49 T magnet (operating at 19.9 MHz for ^1^H), a 10 mm diameter probe, and employs a 90° pulse duration of 2.82 μs (β = 90° = 2.82 μs) along with a 180° pulse duration of 5.14 μs (180° = 5.14 μs), featuring a probe dead time of 7 μs. A detailed description of the data acquisition procedure following these protocols can be found in the Supporting Information.

### ^13^C qSS-NMR Analysis

High-resolution, solid-state ^13^C NMR analyses were conducted by using a Bruker Avance III HD 400 MHz spectrometer operating at a magnetic field strength of 9.4 T, corresponding to a frequency of 400.0 MHz for ^1^H nuclei and 100.5 MHz for ^13^C nuclei. Samples were packed in 4 mm zirconia rotors, and the probe dead time was 15 μs. NMR measurements were performed at 23 °C, which is both the ambient room temperature and the temperature of the spinning air.

For quantitative analysis using the HPDEC sequence, it is necessary to determine the longest *T*_1_ value for the samples. The *T*_1_ values were determined by using an inversion–recovery (IR) pulse sequence. This sequence consists of a 180° pulse, followed by a variable time interval (τ), a 90° pulse, a signal acquisition time (AQ) of 50 ms, and a delay (D1) set to 5*T*_1_. The measurements utilized a 180° pulse with a duration of 8.0 μs, followed by a series of 23 logarithmically spaced τ delays ranging from 0.01 to 1000 s (time τ), a 90° pulse of 4.0 μs (90° = 4.0 μs), AQ = 0.05 s, D1 = 850.0 s, and four scans. The ^13^C signals were decoupled using a Spinal-64 decoupling sequence with a decoupling power (DEC) of 70 W. The sample spinning frequency (SF) was set at 3 kHz. The values of SF, DEC, and D1 were chosen to avoid sample heating.^11^

The *T*_1_ values were obtained through multiexponential fitting of the signals at 34.5 and 30 ppm as a function of the τ values. Figure 1 presents a typical *T*_1_ curve for the signals at 30 and 34.5 ppm for the CB, CL, and chocolate samples. Notably, the *T*_1_ for the signal at 30 ppm is significantly faster than that of the signal at 34.5 ppm.

**Figure 1:**
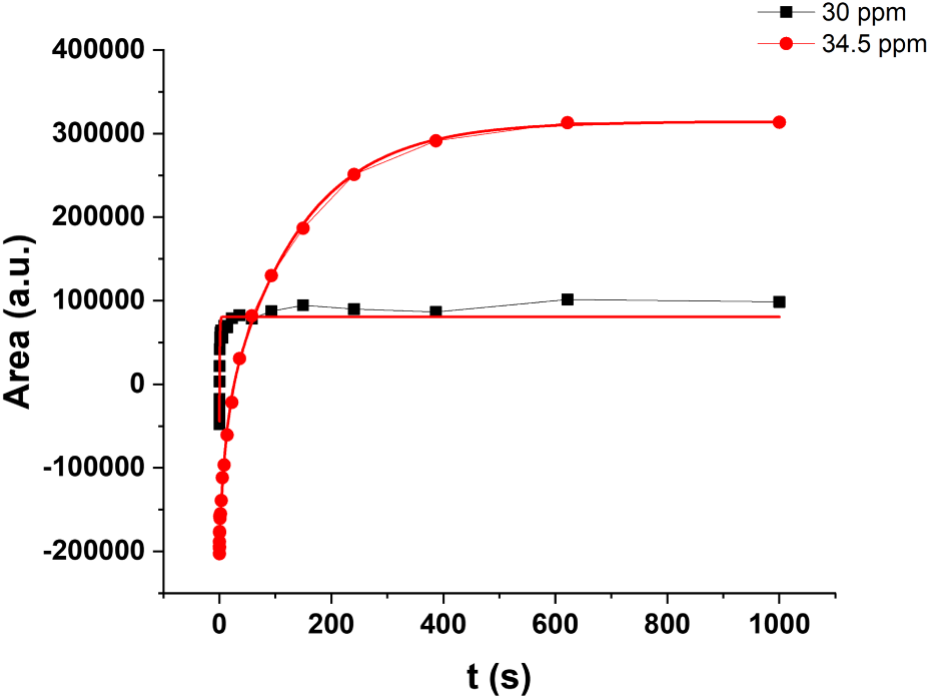
Typical *T*_1_ curve for signals at 30 ppm (black square) and 34.5 ppm (red circle) for cocoa butter (CB), cocoa liquor (CL), and chocolate samples obtained with the inversion recovery (IR) pulse sequence.

Table 1 displays the *T*_1_ values and relative amplitudes for the cocoa butter (CB_1) and chocolate (D70_1) samples, as obtained through multiexponential fitting. For both samples, the *T*_1_ values with the highest amplitude were recorded at 156 and 158 s for the signals at 34.5 ppm and 0.36 and 0.66 s for the signal at 30 ppm.

**Table 1:** *T*_1_ Values and Relative Amplitudes for the Cocoa Butter (CB_1) and Chocolate (D70_1) Samples Determined by Multiexponential Fitting^a^

	Cocoa butter		Dark chocolate	
	*T*_1_ (s)	Amplitude (a.u.)	*T*_1_ (s)	Amplitude (a.u.)
34.5 ppm	0.30	0.05	***	***
	18.8	0.36	29.6	0.57
	**156.0**	**0.59**	**158.0**	**0.43**
30 ppm	**0.36**	**0.67**	0.19	0.28
	20.0	0.33	**0.66**	**0.63**
	***	***	6.53	0.09

a The highest *T*_1_ values are highlighted in bold. *** No *T*_1_ value was detected.

The samples were analyzed using the HPDEC sequence with a recycle delay (D1) set to be equal to or greater than 5*T*_1_ when using a 90° pulse angle (β = 90°). Alternatively, when a lower β value was employed, a significantly shorter D1 of approximately *T*_1_/7 was used. Quantitative measurements were conducted using β = 90°, AQ = 0.05 s, D1 = 850 s, four scans, a DEC of 70 W, and an SF of 3 kHz. Additional measurements were taken with a low β value ranging from 10° to 30°, utilizing D1 = 25 and 128 scans. The spectra acquired through the conventional qNMR method will be referred to as the 90° method (90M) and the low flip angle method (LAM).

All ^13^C SS-NMR signals were processed by using a line broadening of 20 Hz, followed by Fourier transformation and manual phase correction. The area of the solid signals at 34.5 ppm was integrated from 38 to 31.7 ppm, whereas the area for the liquid signal at 30 ppm was integrated from 31.7 to 29 ppm.

### Sample Tempering

The samples underwent thermal treatment (tempering), and the solid fat content (SFC) was determined using the ISO direct and indirect methods, as well as the proposed technique based on ^13^C quantitative solid-state nuclear magnetic resonance (qSS-NMR). The thermal treatment was performed in a Laix dry bath, model PDB-6 (Germany). The samples were maintained at 60 °C for 30 min, followed by 90 min at 0 °C, then 40 h at 26 °C, again 90 min at 0 °C, and finally at 23 °C for 60 min. It is important to note that the final step of the thermal treatment involves recording the temperature, which was chosen to be 23 °C, as this corresponds to the temperature used for the ^13^C qSS-NMR measurements.

## Results and Discussion

### Assignment of the ^13^C SS-NMR Spectra of the CB Sample

Figure 2 shows the HPDEC ^13^C SS-NMR spectra of the CB sample in both liquid (a) and solid (b) phases, which provide information about all carbon types present within the CB TAGs. Figure 2a displays the ^13^C spectrum of a CB sample at MAS with a spinning frequency of 10 kHz. At this spinning frequency (SF), the sample temperature rises to approximately 36 °C, which is sufficient to melt the CB, resulting in spectra that exclusively reflect the liquid phase of the sample.^11,29^ Conversely, in Figure 2b, the spectrum of the same CB sample, acquired at a MAS frequency of 3 kHz, does not significantly raise the sample temperature; thus, the resulting spectrum captures signals from both the liquid (i) and solid (ii) phases, observed at approximately 30 and 34.5 ppm, respectively.^11,29^ The broad solid signal at 34.5 ppm (ii) is attributed to the CH_2_ groups configured in a rigid trans arrangement typical of crystalline solid-state structures.^30,31^

**Figure 2:**
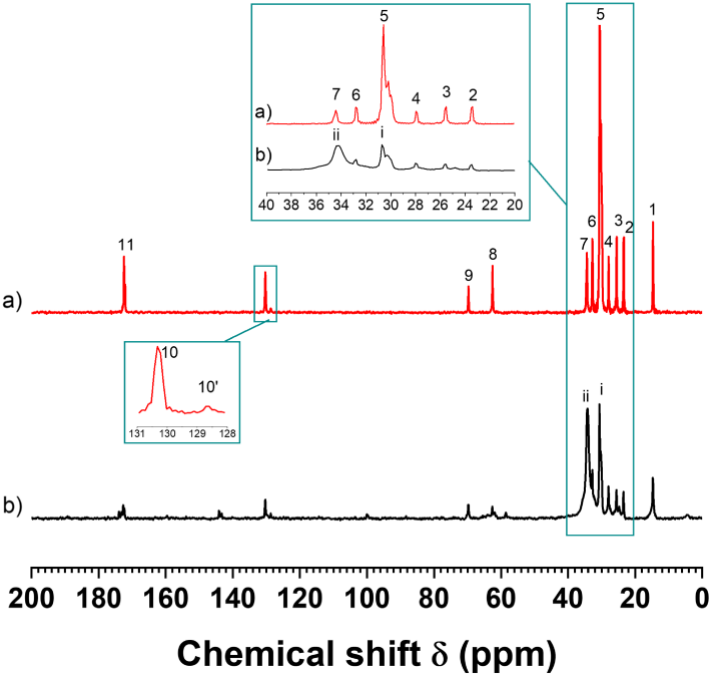
^13^C SS-NMR spectra of a cocoa butter (CB) sample acquired with the HPDEC pulse sequence at spinning frequencies (SF) of 10 kHz (a) and 3 kHz (b). Spectrum (a) represents only the liquid phase, while spectrum (b) includes both liquid and solid phases.

The signals 1–11 depicted in Figure 2a correspond to the carbon atoms within TAG molecules from the CB sample. In general, the fatty acid composition of a CB sample comprises approximately 36% stearic acid (S), 33% oleic acid (O), 26% palmitic acid (P), and 3% linoleic acid (Ln).^18,32,33^ These fatty acids collectively account for over 90% of the fatty acids present in the TAGs of the CB samples. Consequently, the predominant TAGs identified in CB samples include 1-palmitoyl-2-oleoyl-3-stearoyl-glycerol (POS), which constitutes around 37%, 1,3-distearoyl-2-oleoylglycerol (SOS), representing approximately 31%, and 1,3-dipalmitoyl-2-oleoyl-glycerol (POP), which comprises roughly 23%.^18,32,33^ Figure 3 demonstrates the molecular structure of the POS molecule, highlighting the three major fatty acids as illustrative examples of such structures.

**Figure 3:**
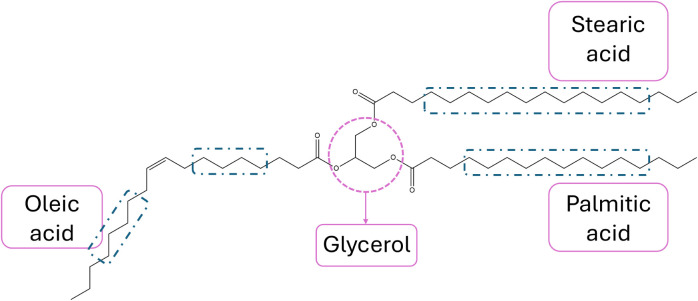
Structure of 1-palmitoyl-2-oleoyl-3-stearoyl-glycerol (POS), one of the major triacylglyceride (TAG) molecules present in cocoa butter (CB).

The ^13^C signal 1 at approximately 15 ppm and the signals 2–7, from 23 to 34.5 ppm, correspond to the terminal CH_3_ and all CH_2_ groups of the various fatty acids, respectively. The signals 8 and 9 at 62 and 70 ppm are assigned to glycerol carbons 1 and 3 and carbon 2, respectively. The peak 10 at 130 ppm is assigned to the double bond carbons 9 and 10 of oleic acid. The peaks at 10 and 10’, highlighted in the inset between the spectra, represent the oleic acid carbons, and the signal 10’ corresponds to the double bond carbons 9 and 12 of linoleic acid, which account for approximately 3% of the fatty acids. The oleic acid signal overlaps the carbons 10 and 13 of linoleic acid signals at 130 ppm. The signal at 172 ppm is assigned to the carboxyl carbons.^34^

The top inset of Figure 2a illustrates the expansion of the signals from the CH_2_ groups, which range from 20 to 40 ppm and will be referenced in the subsequent SFC analysis. The signals identified as 2 (23 ppm), 3 (25 ppm), 6 (32 ppm), and 7 (34.5 ppm) correspond to the CH_2_ groups of carbons ω2, β, ω3, and α, respectively. Additionally, signal 4 (28 ppm) is attributed to the allylic carbons 8 and 11 of oleic acid. The strong peak at signal 5 (30 ppm) is associated with the remaining CH_2_ groups in the inner structures of the fatty acids. These groups, denoted as the (CH_2_)_*n*_ carbons and highlighted by rectangles in Figure 3, represent the 12 carbons in S (from 4 to 15), the 10 carbons in P (from 4 to 13), and the 8 carbons in O (from 4 to 7 and from 12 to 15).^34^

### Quality of ^13^C SS-NMR Spectra Acquired with 90M and LAM Methods

The spectrum shown in Figure 2b, characterized by a high signal-to-noise ratio (SNR), was acquired with β = 90°, D1 = 250 s, and 128 scans, totaling over 7 h. In contrast, a significantly shorter measurement time, resulting in a lower SNR, was employed for both qualitative and quantitative analyses.

Figure 4a presents the spectrum of a chocolate sample, exhibiting an SNR of 19. This was acquired in approximately 1 h using the following parameters: β = 90°, D1 = 850 s, and four scans, referred to as the 90 method (90M). This measurement is notably quicker compared to other quantitative measurements using ^13^C SS-NMR, such as the measurement of lignin products, which requires 2000 scans, D1 = 250 s, and up to 6 days to achieve an acceptable SNR.^10^ The rapid measurement time for the chocolate samples can be attributed to the intense peaks resulting from the sharp lines of TAG molecules, along with the presence of multiple carbons exhibiting the same chemical shift. The SNR obtained within 1 h was adequate for determining the areas of the signals at 30 and 34.5 ppm, which are essential for solid fat content (SFC) measurements.

**Figure 4:**
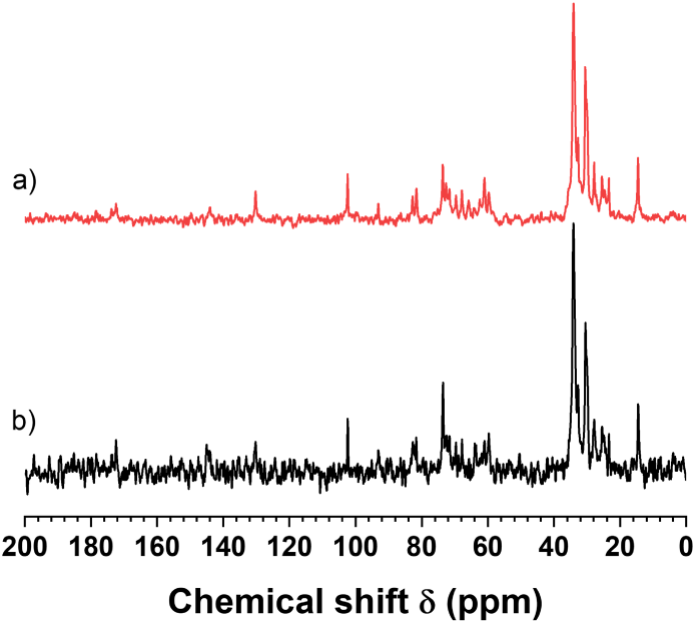
^13^C NMR spectra of chocolate sample D70_2: (a) acquired with β = 90° method (90M) using D1 = 850 s and only four scans, and (b) acquired with the low-angle method (LAM) with β = 23°, D1 = 25 s, and 128 scans. Both spectra were acquired in approximately 1 h. The signal-to-noise ratio (SNR) for 90M and LAM was 19 and 42, respectively.

The signals of non-TAG compounds, such as sucrose (SUC), exhibit a significantly lower SNR (Figure 4a), which complicates qualitative and quantitative analysis. To improve the SNR while maintaining the same measurement time of 1 h, the spectrum was acquired using a shorter recycle delay than *T*_1_, along with a low flip angle (LAM method). This approach is commonly employed to enhance SNR in solution NMR.^12^ Figure 4b presents the spectrum of the same chocolate sample obtained via the LAM method, with β = 23°, D1 = 25 s, and 128 scans. This spectrum demonstrates a notably higher SNR = 42, exceeding twice that achieved with the 90M method. The D1 in the LAM method was set to 25 s to ensure it exceeds the minimum recycle delay required to prevent sample heating due to radiofrequency irradiation, which could potentially melt the TAG molecules.^11^

An unexpected finding was the relative area of the signals at 34.5 and 30 ppm in the LAM spectrum (Figure 4b) compared to that obtained with 90 M (Figure 4a). Given the substantial difference in *T*_1_ for these signals (Figure 1 and Table 1) and the D1 = 25 s used in LAM, a significant reduction in the relative area of the signal at 34.5 ppm was expected.

Upon calculating the initial intensities (time domain) or the areas (frequency domain) for signals at 34.5 and 30 ppm using eq 1,^12^ it became evident that the reduction of solid signals is not as pronounced as initially anticipated. Equation 1 determines the intensity of an NMR signal (*M*_*xy*_) based on the magnetization at thermal equilibrium (*M*_0_), β, D1, and *T*_1_ values. Figure 5 illustrates the calculated intensities (*M*_*xy*_/*M*_0_) for both solid and liquid signals of cocoa butter (Figure 5a) and a chocolate sample (Figure 5b) as a function of β, utilizing D1 = 25 s and the weighted average *T*_1_ derived from the values in Table 1.

1 $$M_{xy}^{\mathrm{init}}=M_{0}\frac{1-\exp\left(-T/T_{1}\right)}{1-\cos\left(\beta \right)\exp\left(-T/T_{1}\right)}\sin\left(\beta \right)$$

**Figure 5:**
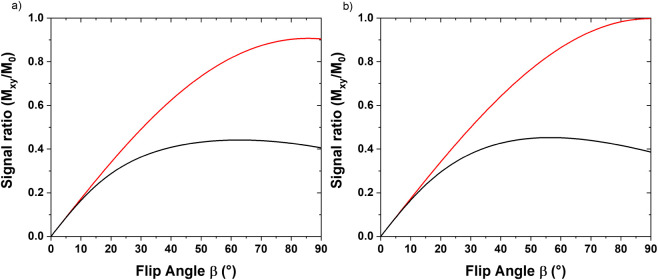
Initial intensities of the transverse magnetizations (*M*_*xy*_) for the signals at 30 ppm (red line) and 34.5 ppm (black line), calculated with eq 1 for β values from 0 to 90, D1 = 25 s, and weighted average *T*_1_ (Table 1) for cocoa butter (CB) (Figure 5a) and chocolate (Figure 5b) samples.

where *M*_*xy*_ is the transverse magnetization, *M*_0_ represents the magnetization at thermal equilibrium, β means the flip angle, and *T* accounts for the repetition time (D1 + AQ).

Figure 5 shows that the difference in initial intensities between the time-domain signals at 30 and 34.5 ppm is not as pronounced as initially anticipated for low β values. Additionally, Figure 5 demonstrates that the disparity between these two signals diminishes as β values decrease, becoming negligible for β ≤ 10°. The *M*_*xy*_/*M*_0_ intensities for the 30 and 34.5 ppm signals in the CB sample were 0.173 and 0.166 for β = 10°, 0.399 and 0.287 for β = 20°, and 0.499 and 0.378 for β = 30°. Similar patterns were observed in the CL and chocolate samples. The relative errors between the two intensities were approximately 4%, 13%, and 25% for β = 10°, 20°, and 30°, respectively. The SFC calculated using the relative areas of the signals at 30 and 34.5 ppm (Figure 3), measured in the 90M and LAM spectra at β = 23°, were 63.1 and 60.4. This aligns with the intensity differences calculated using eq 1, as shown in Figure 4. Although employing LAM with β ≤ 10° reduces the relative error in the area of the two signals, it also diminishes the SNR and offers no clear advantages over the 90 M sequence.

The relative areas of the signals in both methods are maintained not only for the sample illustrated in Figure 4 but also for all of the CB, CL, and chocolate samples analyzed (Figure 6). Consequently, the spectra obtained using the LAM method were utilized for all of the ^13^C SS-NMR analyses, including the SFC measurements.

**Figure 6:**
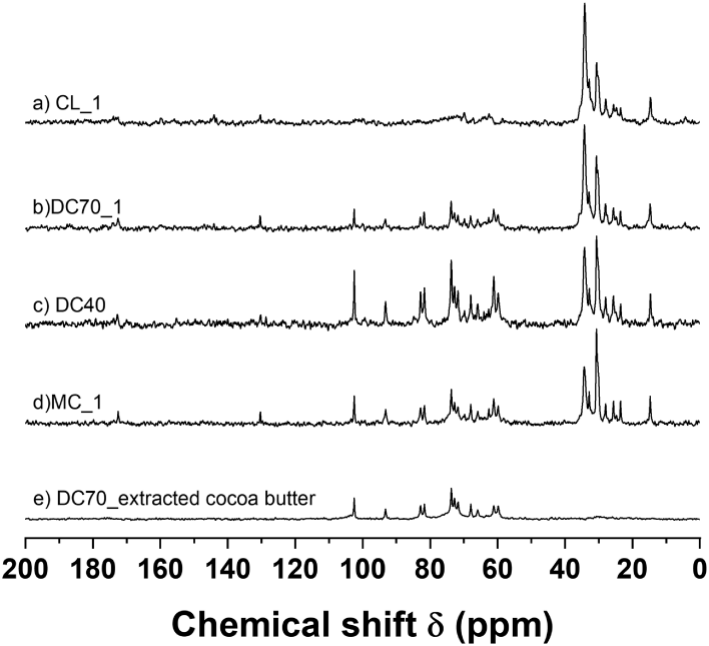
^13^C SS-NMR spectra of cocoa derivative products: (a) cocoa liquor (CL), (b) dark chocolate (DC) with 70% cacao (DC70), (c) dark chocolate with 40% cacao (DC40), (d) milk chocolate (MC), and (e) dark chocolate with 70% cacao without cocoa butter (DC70_extracted cocoa butter).

### Characterization of ^13^C Spectra of Cocoa Liquor and Chocolates

Cocoa liquor (CL) and confectionery chocolates contain various components derived from the cocoa bean, along with added ingredients such as sucrose (SUC) and powdered milk. These products can be detected using ^13^C SS-NMR, particularly when they are present in high concentrations and their signals do not overlap with those of the cocoa bean.

Figure 6 displays the spectra of various cocoa products using the LAM sequence. Figure 6a presents the spectrum of the cocoa liquor (CL_1). Figure 6b,c illustrates the spectra of dark chocolates (DC70_1 and DC40) with cocoa contents of 70% and 40%, respectively. Figure 6e depicts the spectrum of milk chocolate (MC_1). Additionally, Figure 6e displays the ^13^C spectrum of the DC70_1 sample after extracting the cocoa butter (CB) with chloroform, highlighting only the nonfat components. These spectra were obtained using the LAM sequence due to their higher SNR compared to those obtained with the 90 M sequence.

The CL_1 spectrum closely resembles the CB spectrum (Figure 2b), suggesting that the main component of this CL is CB triacylglycerides. Additionally, some minor signals, unrelated to CB and attributed to carbohydrate and protein constituents, can be observed in this spectrum above 50 ppm.

The ^13^C SS-NMR spectra of the chocolate samples (Figure 6b–d) exhibit several distinct peaks, in addition to those corresponding to the triacylglycerides (TAGs) spectra, which have been assigned to the carbon atoms of sucrose (SUC). SUC is commonly added to chocolates as a body agent to enhance sweetness and improve consumer acceptance. The chemical shift signals for SUC, approximately 103, 93, 83, and 82 ppm, do not overlap with those of other compounds and can be utilized for semiquantitative analysis. For quantitative analysis, it is necessary to use an extended recycle delay due to the longer *T*_1_ of the sugar signal. As illustrated in Figure 6b–d, the SUC signals are more pronounced in the dark chocolate containing 40% cocoa (DC40), followed by the milk chocolate (MC) and dark chocolate with 70% cocoa.

The ^13^C SS-NMR spectra can be used to estimate the ratio of TAGs to sucrose (TAGs/SUC). This ratio is calculated using the areas of the signals at approximately 34.5 and 30 ppm, which are associated with TAGs, and the SUC signals that do not overlap with signals from other components. The calculated TAGs/SUC ratios for the spectra of DC70, MC, and DC40 (Figure 6) were found to be 8.3, 3.3, and 1.7, respectively. Consequently, ^13^C SS-NMR can effectively identify SUC as the predominant nonfat component in chocolates.

Figure 6e illustrates the spectrum of the DC70_1 sample following the extraction of the TAG, revealing only the SUC signals. Notably, this spectrum lacks signals from 28 to 35 ppm, which is critical for calculating the solid–liquid ratio of the TAG, commonly referred to as the solid fat content (SFC). Therefore, the ^13^C SS-qNMR method can be effectively employed to determine SFC in cocoa products, such as chocolates, that may contain significant amounts of nonfat solids.

### Determination of the Solid Fat Content of Cocoa Products Using ^13^C qSS-NMR

The solid fat content (SFC) is a crucial factor that affects both the quality of chocolate and consumer acceptance. SFC plays a significant role in determining the organoleptic properties of the final product, which include characteristics such as snap, hardness, shine, and mouthfeel. The mouthfeel, especially the melting characteristic, is closely related to the solid fat content and contributes to sensory evaluations and flavor perception. For instance, when the SFC is high at 37 °C, it can result in incomplete melting, which in turn produces a waxy mouthfeel.^13,35–37^

Several physical techniques can be employed to determine the solid fat content (SFC), including TD-NMR, differential scanning calorimetry (DSC), dilatometry, infrared spectroscopy, ultrasound, and various computational methods.^27,28,38–52^ Among these, ^1^H TD-NMR, direct and indirect methods, has become the standard method in both industry and academia for measuring SFC values.^27,28^ These methods use the free induction decay (FID) signals and are conducted on benchtop NMR instruments.^27,28^ All samples were analyzed using both the direct and indirect ISO methods.^27,28^

The SFC analysis, using both ^13^C qSS-NMR methods (90M and LAM), quantifies SFC by calculating the ratio of the areas of the signals corresponding to the solid and liquid phases at approximately 34.5 and 30 ppm, respectively.^11,30,31^ Both signals are assigned to the same CH_2_ groups situated at the center of the fatty acid structure (Figure 2) within their respective phases.^30,31^

Consequently, the ^13^C NMR analysis excludes data from glycerol carbons, double-bonded carbons, CH_2_ groups adjacent to methyl and carboxyl groups, and terminal CH_3_ groups, thereby differentiating it from standardized TD-NMR methods. The TD-NMR techniques use solely the ratio of the intensities between solid and liquid signals. In contrast, the ^13^C qSS-NMR analysis incorporates both chemical and physical properties.

The SFC of cocoa butter, cocoa liquor, and chocolate samples was calculated using both ^13^C methods, specifically by analyzing the area of the peaks at 30 (*A*_*i*_) and 34.5 ppm (*A*_*ii*_), obtained from the integration of these signals in the ^13^C qSS-NMR spectra. It is essential to highlight that the signal at 34.5 ppm overlaps with signals 6 and 7 observed in the liquid-state spectra (as shown in the inset of Figure 2). Therefore, the combined areas of these two overlapping signals must be subtracted from *A_i_*; this adjustment ensures that the area utilized in the SFC calculations accurately reflects the solid state of the (CH_2_)_*n*_ groups exclusively. The signal area at 34.5 ppm (*A*_*ii*_) is adjusted to *A*_*C*_ by subtracting the areas of peaks 3 (*A*_3_) and 2 (*A*_2_), as outlined in eq 2. These signals correspond to areas 6 and 7 (Figure 2). Although some spinning sidebands are present in the spectra, they do not occur within the spectral region (38–29 ppm) used in the SFC analysis.

The SFC is subsequently calculated using the ^13^C qSS-NMR data, as depicted in eq 3.

2 $$A_{C}=A_{ii}-\left(A_{3}+A_{2}\right)$$

3 $$\mathrm{SFC}\left(\%\right)=\frac{A_{C}}{A_{C}+A_{i}}$$

where *A*_*C*_ represents the corrected area of the solid, *A_ii_* is the signal area at ∼34.5 ppm, *A*_3_ and *A*_2_ are the signal areas at ∼26 ppm and ∼23 ppm, respectively, and *A_i_* is the signal area at ∼30 ppm.

### Comparison of the SFC Values Obtained Using ^13^C and ISO Methods

The SFC values obtained from both ^13^C qSS-NMR methods were compared to those obtained through both direct and indirect ISO protocols. The samples underwent analysis following thermal treatment, as outlined in the ISO procedures. This thermal treatment aims to erase the thermal history of the sample, allowing it to crystallize under controlled conditions, which leads to homogeneous crystallization.

Figure 7 presents the SFC values for the cocoa butter (CB), cocoa liquor (CL), dark chocolate (DC), and milk chocolate (MC) samples, measured using direct (black squares), indirect (red circles), and ^13^C methods for both 90M (blue triangles) and LAM (green diamonds) sequences. The data are categorized into five groups: Group 1 consists of pure CB samples; Group 2 includes CL samples; Groups 3 and 4 feature DC samples with 70% and 60% cocoa content, respectively, while Group 5 comprises the MC samples.

**Figure 7:**
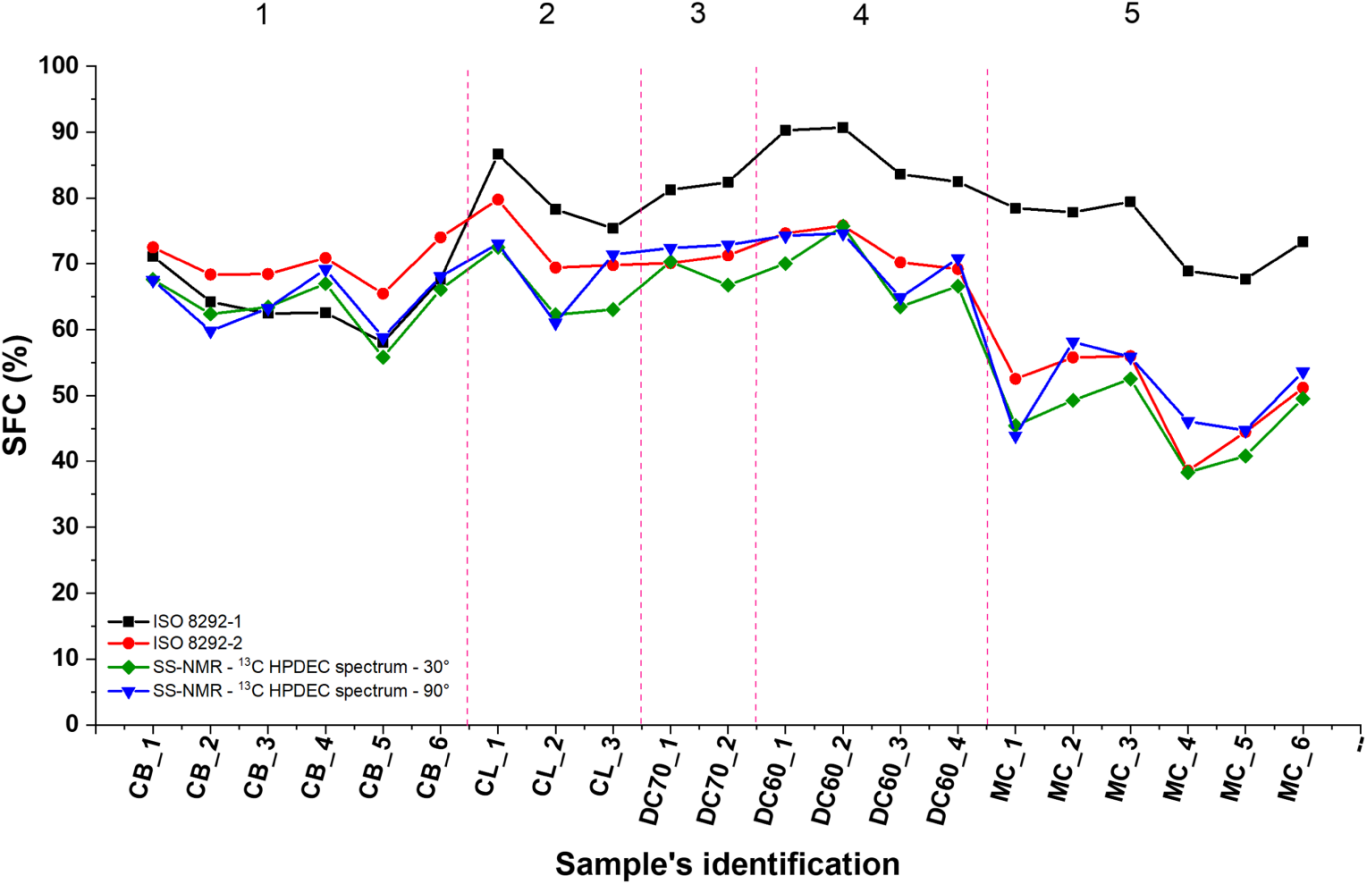
Comparison of the solid fat content (SFC) values obtained through different methods: direct method (black squares), indirect method (red circles), ^13^C qSS-NMR 90M (blue triangles), and the low-angle method (LAM) (green diamonds).

Figure 7 demonstrates that the four methods produce similar results for the CB sample (Group 1), which consists solely of TAGs. In contrast, the CL samples are made exclusively of fermented, roasted, and milled cocoa beans. The spectrum of the CL_1 sample (Figure 6a) reveals a reduced quantity of nonfat solid components, resulting in comparable outcomes across the four methods for this group (Group 2). Notably, the direct method yields the highest SFC value for the CL samples due to the small quantity of nonsolid fats. For Groups 3 to 5, SFC values remain consistent between the indirect methods and the two ^13^C methods, as they are unaffected by nonfat solid contents. However, the direct method shows higher SFC values for these groups, attributed to the larger amounts of nonfat solid contents (Figure 6b–d). Specifically, milk chocolates (Group 5) display lower SFC values, according to both the indirect and ^13^C NMR methods, compared to the other groups, due to the inclusion of powdered milk, which contains 4% to 5.2% milk fat and exhibits lower SFC than CB.

It is noteworthy that the SFC values of all samples, obtained through ^13^C qSS-NMR using both methods, are closely aligned with the SFC values determined by the indirect method, which is recognized as the most accurate TD-NMR method.^45^ This observation implies that ^13^C qSS-NMR may also demonstrate accuracy. However, the accuracy and precision, along with other performance metrics, of the ^13^C SS-qNMR methods still require further investigation, including systematic validation across a range of sample types and concentrations, to confirm the applicability of the method for various analytical settings.

The data shown in Figure 7 present high correlation values, considering the SFC values obtained from the indirect method as the reference standard. The LAM and 90M methods exhibit a positive linear relationship, with Pearson correlation coefficients (*r*) of 0.97 and 0.91, respectively, and both were found to be statistically significant (*p* < 0.05).

### Characterization of Crystalline Forms of TAGs in Cocoa Products

The TAGs of CB can crystallize into as many as six distinct polymorphs, which depend on the crystallization temperature and the duration of cooling.^11,33,53^ These polymorphic forms can be identified using ^13^C SS-NMR by examining the variations in the chemical shifts of the signals associated with methyl, methylene, glycerol, double bond, and carboxyl groups.^11,30,31^

For each polymorphic form, it is observable that distinct physicochemical properties, including variations in stability and melting point, influence the material characteristics. For chocolate production, the crystalline structure V (β) is preferred due to its optimal melting-in-the-mouth behavior (associated with its melting temperature), as well as its snap, resistance to fat bloom, and texture.^18,54^

All the samples examined, including those displayed in Figures 2, 4, and 6, exhibit a chemical shift at 34.5 ppm, which is attributed to the β polymorph, recognized as the most thermodynamically stable form.^33,53^ Other forms have been observed only during the initial stages of the crystallization process.

## Conclusions

The results indicate that both ^13^C qSS-NMR methods are effective for determining the solid fat content (SFC) values of cocoa products (with or without nonfat solids). These methods also enable the characterization of the crystalline polymorphs of triacylglycerides (TAGs) and the major nonfat solids, such as sucrose. The SFC measurements obtained through ^13^C NMR methods are based on the physical and chemical properties of the TAG molecules, as opposed to the standardized protocol, which relies solely on the physical distinction between the solid and liquid phases. Furthermore, the ^13^C methods can be applied to assess similar properties in other fat-containing foods, including butter, margarine, lard, and tallow products. Consequently, the ^13^C qSS-NMR serves as a versatile multianalyte tool that is beneficial for the research and development of new cocoa-based products, as well as for industrial quality control (QC) and quality assurance (QA), and for regulatory agencies to ensure the integrity and composition of cocoa products.

## Supplementary Material



## Notes

Detailed information on how the established ISO protocols determine the SFC values via ^1^H FID signals acquired via TD-NMR (PDF)
