# Supplementary material for: Rapid 13C Solid-State Quantitative NMR Method for Multiple Physical and Chemical Analyses of Cocoa-Based Products: Proof of Concept
Source: Anal Chem. 2025 Sep 7;97(36):19909–17. doi: 10.1021/acs.analchem.5c04122 (PMC12444744; doi:10.1021/acs.analchem.5c04122)
Supplement: Supplementary file 1 [file ac5c04122_si_001.pdf]

# Rapid $^{13}C$ solid-state quantitative NMR method for multiple physical and chemical analyses of cocoa-based products: Proof of concept

Thais Juliana Tobias[1], Priscilla Efraim[2], Tiago Bueno de Moraes[3], Luiz Alberto Colnago[4]*

*[1] São Carlos Institute of Chemistry, University of São Paulo (USP), Av. Trabalhador São-carlense 400, 13660-970, São Carlos, SP, Brazil*

*[2] Department of Food Engineering and Technology, University of Campinas (UNICAMP), St. Monteiro Lobato 80, 13083-862, Campinas, SP, Brazil*

*[3] Department of Biosystems Engineering, Luiz de Queiroz College of Agriculture, University of São Paulo (USP), Av. Pádua Dias 11, 13418-900, Piracicaba, SP, Brazil.*

*[4] Embrapa Instrumentation, Rua XV de Novembro 1452, São Carlos, SP, 13560-970, Brazil.*

*Corresponding author (L.A. Colnago)

E-mail addresses: luiz.colnago@embrapa.br

**Supporting Information**

## Detailed information on how the established ISO protocols determine the SFC values via $^1$H FID signals acquired via TD-NMR

The direct SFC measurement (Figure S1) uses the FID intensity at 11 μs ($S_{11}$), at 70 μs ($S_{70}$), and f-factor (a correction factor due to the dead time of the instrument) for the calculation, using Equation S1. The intensity $S_{11}$ corresponds to the total $^1$H concentration in the sample due to the solid and liquid components, and $S_{70}$ to the $^1$H concentration for the liquid components [1]. The determination of the f-factor must be performed prior to the acquisition of the FID signals, following the ISO 8292-1 protocol. [1]

$$SFC\ (\%) = \frac{f * (S_{11} - S_{70})}{f * (S_{11} - S_{70}) + S_{70}} * 100 \qquad \text{Equation S1}$$

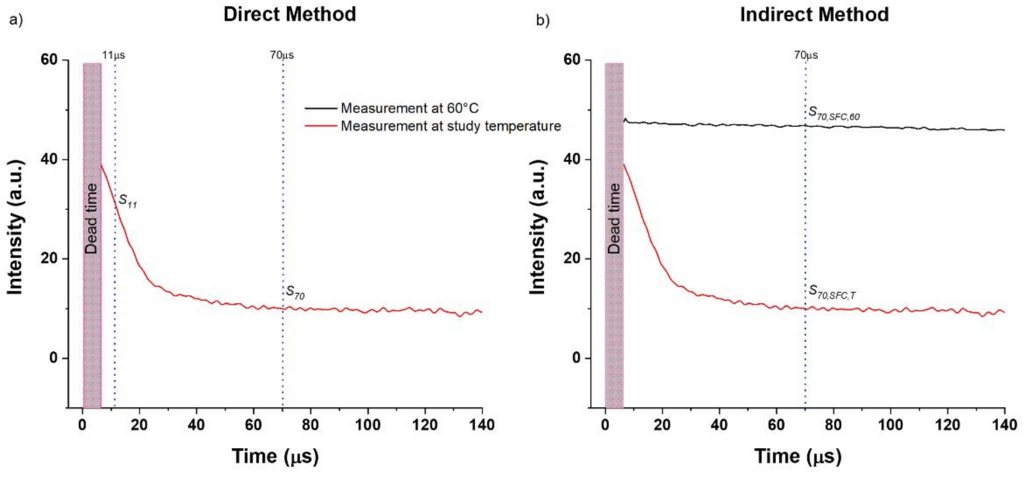


**Figure S1.** FID signals used for the determination of SFC. The direct method (a) uses only the FID signal shown in, at 11 and 70 μs, and the f-factor and Equation

S1. The indirect methods (b) require measurements of the intensity of FID signals for the sample at 60°C and the signal of the same sample at the reading temperature (a). The indirect method also requires the measurement of a reference sample that is liquid in both temperatures. The indirect SFC method is calculated using Equation S2.

The indirect method requires measurements of the FID signals at 70μs of the samples in two temperatures: one at 60°C, which guarantees complete melting of the samples, and one measurement at the reading temperature, which contains both solid and liquid components. [2]

Figure S2a shows the FID signal of a CB sample at 60°C that has only the liquid component, while Figure S2b shows the FID signal of a CB sample at the reading temperature, meaning the temperature which the SFC is determined (in this example, 23°C), which shows both solid and liquid components. The FID intensities are measured only at 70 μs, without considering the solid signal, and, therefore, do not require a probe with short dead time. The method also requires the measurement of a reference sample that is liquid in both temperatures to correct the variation of the NMR signal due to the Boltzmann effect. The SFC value is calculated using the FID intensity at 70 μs obtained in the four measurements according to Equation S2. [2]

$$SFC_T\ (\%) = \left(1 - \frac{S_{70,ref,60} * S_{70,\,SFC,\,T}}{S_{70,\,SFC,\,60} * S_{70,\,ref,\,T}}\right) * 100 \qquad \text{Equation 2}$$

Where $S_{70,ref,60}$ is the intensity of the FID measured at 70 $\mu$s of the reference sample at 60°C; $S_{70,ref,T}$ is the intensity of the FID measured at 70 $\mu$s of

the reference sample at the reading temperature; $S_{70,SFC,60}$ is the intensity of the FID measured at 70 $\mu$s of the test sample at 60°C and $S_{70,SFC,T}$ is the intensity of the FID measured at 70 $\mu$s of the test sample at the reading temperature. [2] As the measurements are performed only at 70 μs, the non-fat components of chocolate, for example, do not interfere in the SFC of the cocoa butter fraction. [2]

## Supplementary References

(1) ISO/CD 8292-1. ISO/CD 8292-1: 2008. Animal and Vegetable Fats and Oils - Determination of Solid Fat Content by Pulsed NMR - Part 1: Direct Method. International Organization for Standartization 2008.

(2) ISO/CD 8292-2. ISO/CD 8292-2:2008 - Animal and Vegetable Fats and Oils - Determination of Solid Fat Content by Pulsed NMR - Part 2: Indirect Method. International Organization for Standartization 2008.
